# The Stimulatory Effect of Cerebral Intraventricular Injection of cNPY on Precocial Feeding Behavior in Neonatal Chicks (*Gallus domesticus*)

**DOI:** 10.1371/journal.pone.0153342

**Published:** 2016-04-07

**Authors:** Guiqian Chen, Feifei Yang, Taofen Wu, Junfang Jiang, Weidong Zhou

**Affiliations:** 1 Institute of Animal Husbandry and Veterinary Science, Zhejiang Academy of Agricultural Sciences, Hangzhou, Zhejiang 310021, China; 2 School of Medicine, Jiaxing University, Jiaxing, Zhejiang 314000, China; 3 Lianyungang Biological Engineering Specialty Secondary School, Lianyungang, Jiangsu 222248, China; 4 College of Animal Science, Zhejiang University, Hangzhou, Zhejiang 310058, China; 5 R&D center, Zhejiang EBVAC Biotech Co., Ltd., Hangzhou, Zhejiang, 310018, China; University of Rouen, France, FRANCE

## Abstract

Neuropeptide Y (NPY) is one of the most potent stimulants of food intake in many animals. Most of the supporting evidence for the effects of NPY has been gathered in mammalian species using porcine NPY. To investigate the effects of NPY on precocial feeding initiation in chicks, we firstly used chicken NPY (cNPY) to study its role in food intake and spontaneous activities in 3-day-old male chicks. Food intake was monitored at different times after intracerebroventricular (ICV) injection of cNPY (2.5, 5.0 or 10.0 μg/10 μL) and anti-cNPY antibody (anti-cNPY) (1:9000, 1:3000 or 1:1000 in dilution). cNPY given at different doses significantly increased food intake at 30 min, 60 min, 90 min and 120 min after injection. Chicks treated with 5.0 μg/10 μL of cNPY showed a maximal 4.48 fold increase in food intake comparing to the control at 30 min. There is still more than 2 fold increase in food intake at 120 min after injection of cNPY. Food intake was significantly inhibited by a single ICV injection of anti-cNPY diluted to 1:9000 (60% inhibition), 1:3000 (92% inhibition), and 1:1000 (95% inhibition) at 30 min with 1:1000 being the maximally effective concentration. The inhibitory effects of anti-cNPY (diluted to1:9000, 1:3000, 1:1000) at 120 min post ICV injection were 22%, 42% and 46%, respectively. But ICV of anti-cNPY (1:3000 in dilution) did not block the orexigenic effect of 2.5 μg/10 μL of cNPY. ICV injection of different concentrations of cNPY increases locomotor activity in a dose-dependent manner while ICV anti-cNPY greatly decreased the distance moved by each chick compared to control groups. Taken together, our results demonstrated that cNPY has a promoting effect on chick food intake and locomotor activity, and that endogenous cNPY might play a positive role in regulating precocial feeding behavior in newly hatched chicks.

## Introduction

Neuropeptide Y (NPY) is a highly conserved 36-amino-acid amidated peptide belonging to the pancreatic polypeptide family, and is widely distributed in the central nervous system of various species including fishes, birds and mammals [1, 2] and is associated with the regulation of energy homeostasis and appetite [3–5]. Central administration of NPY is reported to stimulate food intake in chicks [6], rabbits [7], rats [8], mouse [9] and zebrafish [10], and reports also indicated NPY to be functional to drinking responses [7, 11]. However, in other species such as the baboon, neuropeptide Y does not stimulate food intake [12]. The evidence of stimulatory effects of NPY on feeding comes from data on intracerebroventricular (ICV) injection of porcine NPY due to the highly conservation of NPY between avian and mammal. However, reports also indicate that the complete amino acid sequence of centrally administered NPY is required for maximal food intake response [13], suggesting that the complete sequence of NPY may function in a specific manner in certain species, and even a difference of one amino acid, such as NPY between the mammal and avian species, may have a distinct effect of NPY on feeding behavior. Currently there is no information on chicken NPY (cNPY) on food intake in chickens. Elucidating the specific effect of chicken NPY on feeding behavior in chicks will be valuable for us to understand the detailed effects of NPY in food intake in avian species.

Previous studies indicated that NPY is a physiological signal involved in the stimulation of ingestive behavior in rats [15]. Conditions of food deprivation and ingestion induce reciprocal changes in neuropeptide Y concentrations [16]. Increased food intake by NPY is due to an increased motivation to eat [17]. We have previously reported that endogenous cNPY is increased during the stages of embryo development [18, 19], with the increase of cNPY being particularly robust when the embryos are close to hatching, indicating the possible role of endogenous cNPY in induction of food intake in chicks.

We propose that cNPY functions not only on stimulation of food intake in chicks, but also may be beneficial to locomotor activities in order to search for the food. However, currently there is a lack of evidence regarding the role of exogenous cNPY on feeding behavior. In order to reveal the effect of cNPY on precocial feeding behavior, we firstly investigated the effect of cNPY on food intake in free-feeding unrestrained chicks and discuss the results of previous studies using porcine NPY. We also investigated the effect on locomotor activity of cNPY or anti-cNPY antibody (anti-cNPY) post ICV injection. The study will help us understand better on the stimulatory effect of cNPY in neonatal chicks as well as its possible role in precocial feeding behavior.

## Materials and Methods

All experimental protocols in this study received approval from the Institutional Animal Care and Use Committee of Zhejiang Academy of Agricultural Sciences of China.

### Animals

One-day-old male chicks (hy-line) were purchased from a local farm. They were raised in an electrically heated brooder at 28°C room temperature under continuous lighting using fluorescent lamps, and were provided *ad libitum* a commercial grower diet (crude protein: 21%, metabolisable energy: 12.3 MJ/kg) and water. At 3 days of age, the chicks were selected for ICV injection experiments by their body weight, and were divided into several experimental groups with no statistical differences in weight.

Experiment 1: Chicks were divided into 4 groups (14 chicks/group), and each group received ICV injected cNPY at different doses (vehicle, 2.5 μg, 5 μg and 10 μg/10 μL). Food intake was determined at 30 min, 60 min, 90 min and 120 min after the injection. The doses of cNPY and post-injection period were decided based on a previous bird study [1, 6].

Experiment 2: Chicks were divided into 4 groups (10 chicks/group), and each group was ICV injected anti-cNPY working solutions with different dilutions (vehicle, 1:9000, 1:3000, and 1:1000 in dilution). Food intake was determined at 30 min, 60 min, 90 min and 120 min after the injection. Dosage of anti-cNPY and post-injection period were decided according to the study by Ishii et al [20].

Experiment 3: Chicks were divided into 4 groups (12 chicks/group), and each group was ICV injected vehicle, cNPY (2.5 μg), anti-cNPY (1:3000 in dilution), and mixture of cNPY (2.5 μg) and anti-cNPY (1:3000 in dilution) working solutions. Food intake was determined at 30 min, 60 min, 90 min and 120 min after the injection.

Experiment 4: Chicks were divided into 8 groups (8 chicks/group), and each chick in 4 groups was ICV injected cNPY solutions with different doses (vehicle, 2.5 μg, 5 μg and 10 μg), and in another 4 groups was ICV injected vehicle, cNPY (2.5 μg), anti-cNPY (1:3000 in dilution), and mixture of cNPY (2.5 μg) and anti-cNPY (1:3000 in dilution) working solutions, respectively. Moving distance of chick was recorded for 10 min from 5 min to 15 min after injection.

### Injection solution preparation

Chicken NPY (P01882) was synthesized by Sangon Biotech Inc (Shanghai, China). The stock cNPY solution 2 μg/μL was prepared using a Ringer solution (147 mM NaCl, 4 mM KCl, 3 mM CaCl_2_, and 0.1% bovine serum albumin dissolved in MilliQ water). The rabbit antiserum against cNPY (bs-0071R) was purchased from Boisynthesis Biotechnology Co. Ltd (Beijing, China). Evans Blue Dye (EBD) solutions 0.1% and 0.2% were prepared using the Ringer solution. The cNPY solution and anti-cNPY were stored at minus 20°C, and the EBD solutions were stored at 4°C until used for the ICV working solutions preparation.

Working concentrations of cNPY and anti-cNPY working solutions were freshly prepared before each experiment, and dosages were determined based on previous studies [20–22]. Briefly, the concentrations of 1.0 μg/μL, 0.5 μg/μL, 0.25 μg/μL cNPY and 1:1000, 1:3000, 1:9000 anti-cNPY working solutions were prepared by diluting the stock solution using 0.1% and 0.2% EBD solution. The mixed working solution of 0.25 μg/μL cNPY and 1:3000 anti-cNPY was obtained by mixing 0.5 μg/μL, 0.25 μg/μL cNPY and 1:1000 anti-cNPY working solutions in correct proportions.

### ICV injection

ICV injection was performed as reported previously [23]. At 3 days, the chick head was fixed using a stereotaxic ear bars, and the syringe needle was unilaterally (right side) implanted towards the lateral ventricle. The stereotaxic coordinates were: 5–8 mm anterior to the centered ear bars, 0.3–0.5 mm lateral to the midline, and 3–4 mm below the skull surface [6]. This method didn’t appear stressful for the chicks and didn’t affect feeding behavior [24]. All injections were administered using a 15-μL syringe (Hamilton, Reno, USA). The injection volume was 10 μL per chick in all experiments, and the vehicle group was injected with the same volume of 0.1% EBD solution.

### Food intake determination

Immediately after the ICV injection, the chicks were housed individually in cages, and food intake was recorded at 30 min intervals for 2 h. The weight of the feeding trough containing feed was recorded at the start point time and 30 min, 60 min, 90 min, 120 min after the ICV injection, and food intake were calculated accordingly.

### Locomotor activity determination

The chicks were spotted with different colors on their heads to differentiate between the experimental groups, and the colors could be distinguished by Noldus Ethvosion software, which was used to analyze the locomotion of the animals. After delivery of the solutions, four chicks were simultaneously placed into a 400 (L) × 300 (W) × 200 (H) mm paper box containing food on the bottom. Video cameras were positioned over the box to record the chick locomotor activities for 15 min. Moving track and distance were recorded using a computerized tracking system from 5 min to 15 min after injection, and were analyzed by the Noldus Ethvosion platform from Zhejiang Sci-Tech University.

### Histological analysis

After ICV experiments, chicks were deeply anesthetized by intraperitoneal injection using sodium pentobarbital, and euthanized by decapitation. The brain was removed, and was dissected according to the brain atlases the chick [6] and previous reported method [18]. The presence of the EBD in the lateral ventricle was checked, and the data was deleted if the EBD could not be verified.

### Data analysis

All results are presented as the means ± SEM. Statistical significance in food intake and moving distance were assessed by two-way analysis of variance (ANOVA) with time and dose as classification variables, and cumulative moving distance was assessed by one-way ANOVA. When ANOVA showed that dose had a significant effect, comparisons among different dose groups at the same time were performed using Tukey’s multiple comparison tests. *P* < 0.05 was considered statistically significant.

## Results

### Stimulatory effect of cNPY on food intake in free-feeding chicks

**Fig 1:**
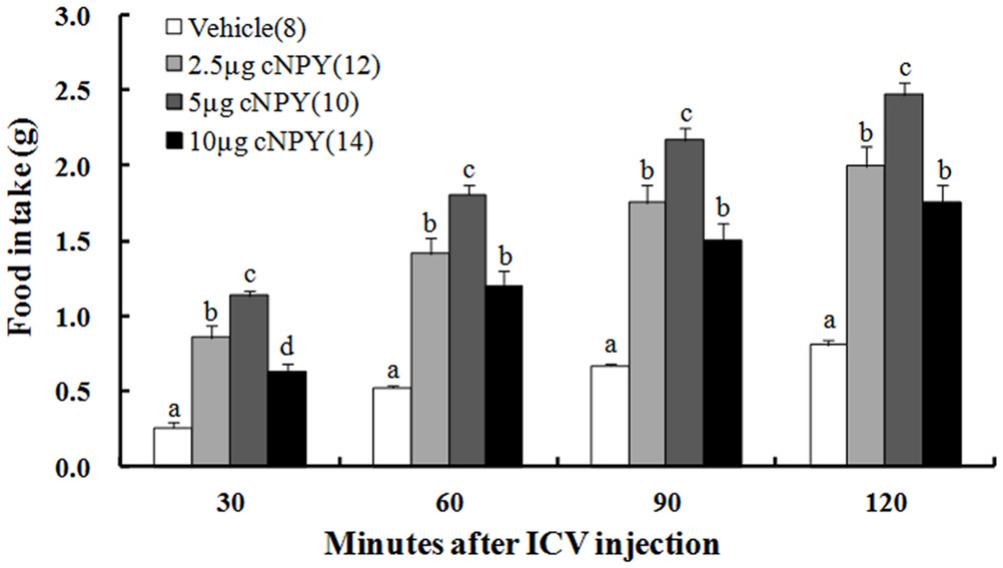
Effects of ICV injection of cNPY on food intake in free-feeding chicks. The numbers of chicks in each group are shown in parentheses. Values shown are presented as the means ± SEM. Columns with different letters are significantly different among groups at each time (*P* < 0.05).

Significant treatment [F (3, 40) = 116.606], time [F (3, 44) = 89.151] and their interaction [F (9, 44) = 1.959] effects were found on food intake (Table 1). At all the times after the injection, all the treatments of cNPY significantly increased food intake than the vehicle group (Fig 1). The doses of 2.5 μg and 10 μg cNPY-treated chicks ate at least twice as much food than the vehicle group, and food intake of 5.0 μg cNPY-treated chicks was greatest among cNPY-treated groups. The stimulatory effects of cNPY on food intake in all doses wore off with time after the injection.

**Table 1:** Statistical significance (*P* values) for effects of time, treatment and their interaction on food intake and moving distance after ICV injection of cNPY and anti-cNPY in free-feeding chicks using two-way ANOVA.

Experiment	Treatment	Time	Treatment	Time × Treatment
**Food intake**	cNPY	0.001	0.001	**0.047**
	anti-cNPY	0.001	0.001	**0.979**
	cNPY and anti-cNPY	0.001	0.001	**0.018**
**Moving distance**	cNPY	0.986	0.001	**0.705**
	**cNPY and anti-cNPY**	**0.663**	**0.001**	**0.914**

### Inhibitory effects of anti-cNPY on food intake in free-feeding chicks

**Fig 2:**
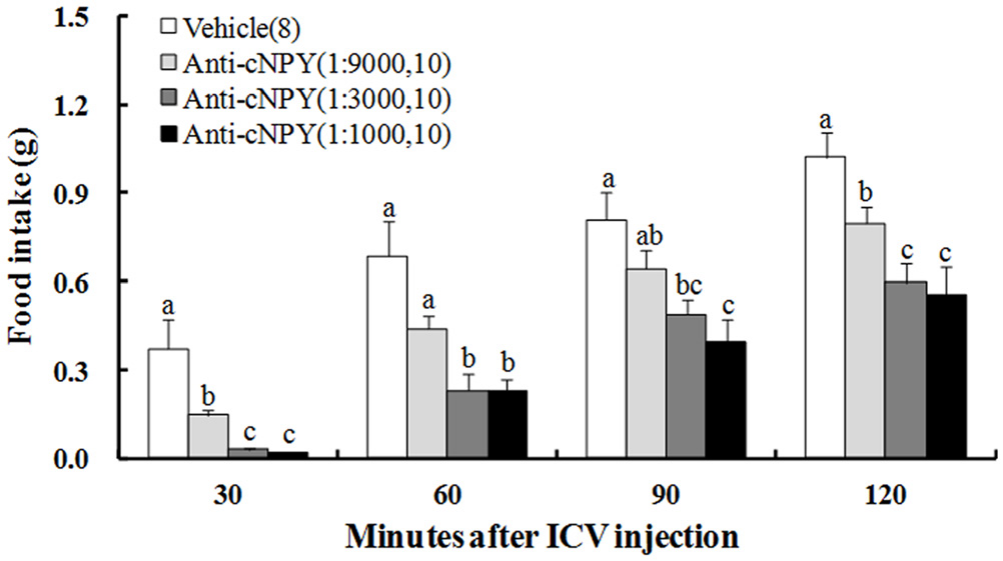
Effect of ICV injection of anti-cNPY on food intake in free-feeding chicks. The numbers of chicks in each group are shown in parentheses. Values shown are presented as the means ± SEM. Columns with different letters are significantly different among groups at each time (*P* < 0.05).

Significant treatment [F (3, 34) = 32.158] and time [F (3, 38) = 61.864] effects were found on food intake, but their interaction [F (9, 38) = 1.959] effect was not significant (Table 1). At all times after injection, food intake was significantly inhibited by a single ICV injection of anti-cNPY solution (Fig 2). Food intake for all concentrations was significantly less than the vehicle group except the 1:9000 dilution at 60 and 90 min, and no difference of food intake between in 1:3000 and 1:1000 dilutions was found. The inhibitory effects of anti-cNPY on food intake between in 1:1000 and 1:3000 dilutions were very similar, and were obviously greater than that in 1:9000 dilution. The inhibitory effects of anti-cNPY on food intake in all dilutions wore off with time after the injection.

### Effects of combining cNPY with anti-cNPY on food intake in free-feeding chicks

**Fig 3:**
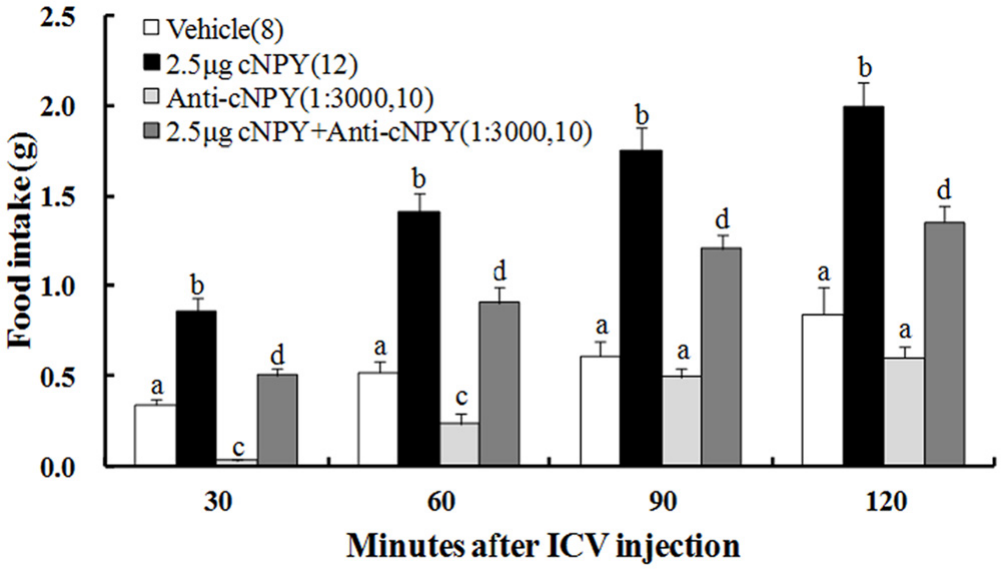
Effect of ICV mixture injection of cNPY and anti-cNPY on food intake in free-feeding chicks. The numbers of chicks in each group are shown in parentheses. Values shown are presented as the means ± SEM. Columns with different letters are significantly different among groups at each time (*P* < 0.05).

Significant treatment [F (3, 34) = 139.559], time [F (3, 40) = 53.373] and their interaction [F (9, 40) = 1.959] effects were observed on food intake (Table 1). Chicks treated with the mixture of cNPY and anti-cNPY ate (0.3–0.4 folds) less food than cNPY-treated chicks but their food intake still more than the vehicle group at all the times after the injection (Fig 3). However, food intake of chicks treated with anti-cNPY at 1:3000 dilution was lower than that of the vehicle group with significant difference at 30 min and 60 min but not at 90 min and 120 min.

### Effects of cNPY and anti-cNPY on locomotor activity in unrestrained chicks

**Fig 4:**
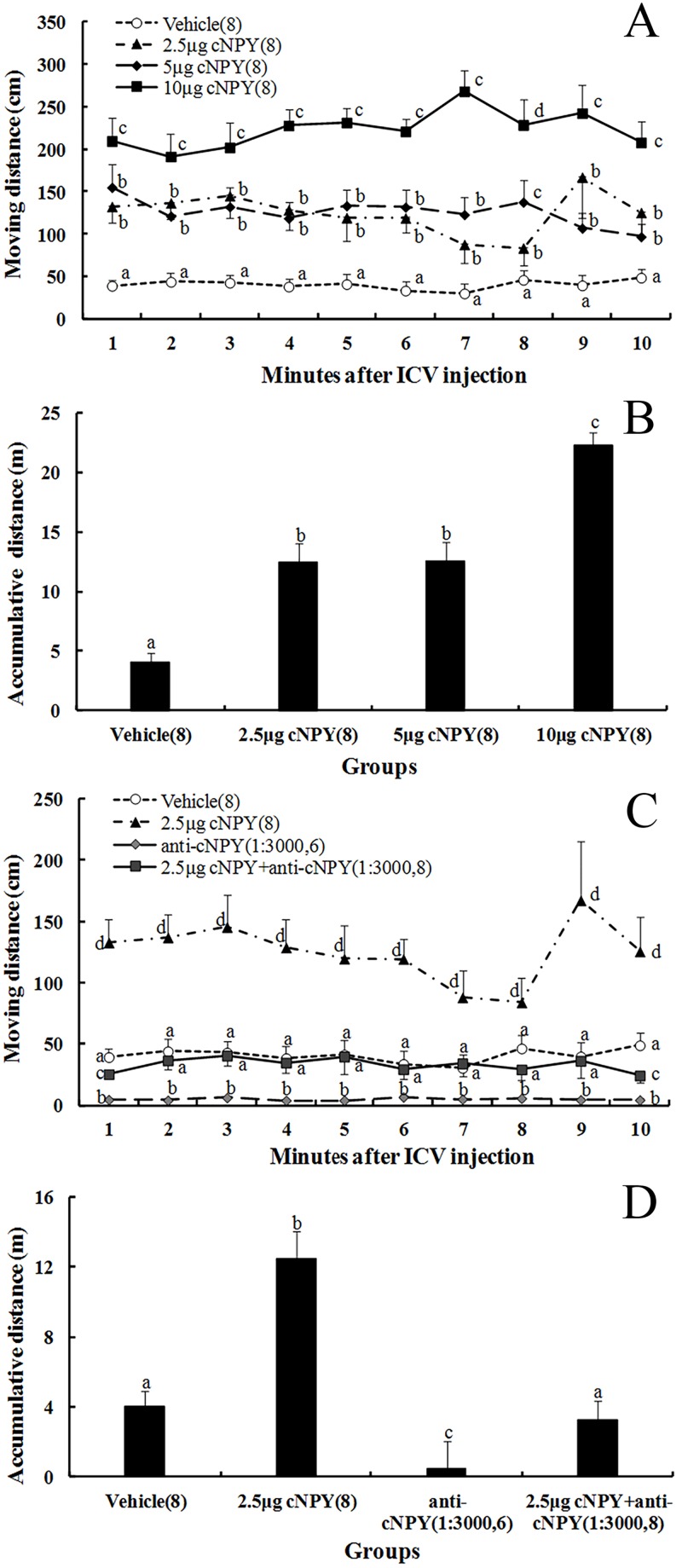
Effects of ICV injection of cNPY (A and B), anti-cNPY and its mixture (C and D) on moving distance and cumulative distance for 10 min in unrestrained chicks. The numbers of chicks in each group are shown in parentheses. Values shown are presented as the means ± SEM. Columns with different letters are significantly different among groups at each time (*P* < 0.05).

The effect of dose [F (3, 26) = 103.748] on distance moved was significant, but the effects of time [F (9, 30) = 0.253] and their interaction [F (27, 30) = 0.834] were not significant (Table 1). All the treatments of cNPY significantly increased distance moved with respect to the vehicle group (Fig 4A and 4B). Distances comparing between 2.5 μg and 5.0 μg cNPY-treated chicks were not significantly different except the 8^th^ min, but were less than that of 10 μg cNPY-treated chicks. The distinctive inducer of cNPY on distances moved seems to be dose-dependent.

**Fig 5:**
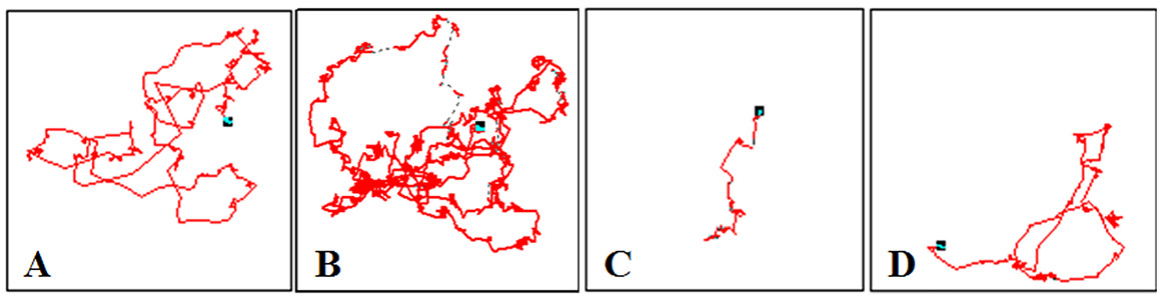
Typical profiles of effects of ICV injection of cNPY and anti-cNPY on moving track and distance for 10 min in unrestrained chicks. A: Vechile (0), B: cNPY (2.5 μg), C: anti-cNPY (1:3000 in dilution), D: cNPY (2.5 μg) + anti-cNPY (1:3000 in dilution).

Significant treatment [F (3, 26) = 102.381] effect was found on distance moved, but the effects of time [F (9, 30) = 0.750] and its interaction [F (27, 30) = 0.645] with treatment were not significant (Table 1). Distance moved in anti-cNPY treated chicks were significantly less than the vehicle chicks and cNPY-treated chicks, and in cNPY and anti-cNPY mixture-treated chicks were significantly increased to levels in the vehicle chicks, but was still significantly less than cNPY-treated chicks (Figs 4C, 4D and 5).

## Discussion

It has previously been shown that endogenous NPY is released in PVN, and hypothalamic prepro-NPY mRNA levels are increased with fasting and normalized by refeeding [25]. NPY is indicated to be one normal physiological signal involved in the stimulation of ingestive behavior in rats [15]. Since NPY is firstly isolated from porcine brain [26], the stimulatory effect of NPY on food intake is studied using the exogenous porcine NPY. The results in the present study found that cNPY significantly increased food intake in 3-day-old male chicks at all experimental doses and times, and the stimulatory effect of 5.0 μg cNPY-treated chicks on food intake was the strongest. However, the 10 μg cNPY-treated chicks ate less food than 2.5 μg cNPY-treated chicks (Fig 1). Similar results are also observed using porcine NPY in 2-days-old Leghorn chicks [22, 27, 28]. These results suggested that cNPY and porcine NPY did not elevate food consumption significantly in a dose-dependent fashion, and trigger similar fashion on food intake in precocial chicks in a similar fashion. In young rabbits, injection of 1, 5 and 10 μg doses of porcine NPY immediately increased in feeding and drinking were evident in a dose-related manner [7], and the similar responses are also observed after porcine NPY injection in young sheep [29], rats [30] and dove [31]. Similar to mammalian NPY, chicken NPY has five receptor subtypes (named Y1-Y6, Y3 receptor is not cloned yet) in the chicken [32]. Among these receptors, the Y1 and Y5 receptors appear to represent the most likely candidates for mediating regulation of feeding behavior and energy homeostasis of NPY in both mammals and birds [33]. The development of NPY receptor subtypes is later than that of NPY [34]. These results suggested that NPY functions in a variable manner depending on age and species.

Previous studies also showed that the effective doses range lies between 0.25–9.0 μg (59–2116 pmol porcine NPY) in more than 2 weeks old chicks [22] and the maximally effective doses in different studies have tended to induce a 2-fold to 4-fold increase in food intake which can be detected at 30–60 min post the injection [22, 28]. In the present study, cNPY stimulated a 2.17 fold to 4.48 fold increase on food intake at 30–120 min post the injection, suggesting that cNPY has considerable stronger and long-term effect on food intake in chicks than that using porcine NPY does. The cNPY and porcine NPY have a sequence difference of one-amino-acid [14], and the Y1 and Y5 receptors of the amino acid identities are about 85% and 77% in chicken when the transmembrane regions were compared to their mammalian orthologues [32, 35, 36]. These may partly explain the different effect of cNPY on appetite in chicks.

Stronger magnitude of cNPY on food intake may be helpful to induce longer-term effect on precocial chicks to search for food when facing food deprivation. Neonatal chicks have to search food independently for their growth and development, and NPY is reported to be associated with motivation to eat [17]. Since they are precocial animals, wide-range of locomotion activity would be helpful for chicks to search for food and feed themselves [37]. Thus, higher magnitude of cNPY at early stages in the neonatal chicks seems to be imperative. Locomotor activities are associated with the feeding behavior [37, 38] and influence ingestive behaviors in rat [39]. In the present study, cNPY significantly elevated distance moved by chicks in a dose-dependent fashion under feeding conditions (Fig 4A and 4B), and the stimulatory effect of 5.0 μg cNPY-treated chicks was the strongest. However, the 10 μg cNPY-treated chicks ate less food than 2.5 μg and 5 μg cNPY-treated chicks (Fig 1). Thus, at the early stage in the neonatal chicks, the stimulatory effect of NPY on locomotor activity may take precedence over food intake.

The results of the exogenous NPY ICV injection confirmed that the hypothalamus may be involved in the NPY-regulation of feeding behavior and energy homeostasis in birds [1, 38]. In the present study, food intake and locomotor activity were significantly inhibited by a single ICV injection of anti-cNPY solution in a dosage-dependent manner in free-feeding unrestrained chicks (Figs 2, 4C and 4D), suggesting that the endogenous NPY signal in the hypothalamus was effectively blocked by its antibody, and may play a positive role in food intake and locomotor activity in the chicks. The inhibitory effect relaxed at first 30 min of anti-cNPY diluted 1:9000 (60% inhibition), 1:3000 (92% inhibition), and 1:1000 (95% inhibition). The inhibitory effect by anti-cNPY antibody (1:3000 and 1:1000 in dilution) seems to be maximal, suggesting that anti-NPY solution diluted 1:3000 was enough to the stimulatory effect of the endogenous NPY in the neonatal chicks. Injection of NPY antisera in mouse suppressed food consumption, and the increase in food intake caused by a 24 h fasting was significantly inhibited by ICV injection 5 μL per mouse of anti-NPY antibody diluted 1:1500 (52% inhibition), 1:4000 (48% inhibition) and 1:8000 (33% inhibition) [20]. These results suggested that the suppressive effect of anti-cNPY seems to be greater in neonatal chicks than that in the mice.

With ICV injection of 2.5 μg cNPY combined with anti-cNPY solution (1:3000 in dilution), chicks ate less food (0.4 folds) than 2.5 μg cNPY-treated chicks but greater than the vehicle and anti-cNPY solution (Fig 3), and moved less distances (0.7 folds) than 2.5 μg cNPY-treated chicks but similar to the vehicle (Fig 4C and 4D). These results indicated that cNPY-induced increment in food intake and locomotor activity was partly blocked by anti-cNPY, and the blocking effect of anti-cNPY on the locomotor activity seems to be greater than that on food intake.

In conclusions, our results demonstrate that cNPY promotes chick food intake and locomotor activity, and that endogenous cNPY might play a positive role in the regulation of the precocial feeding behavior in newly hatched chicks.
